# Inter-software and inter-scan variability in measurement of epicardial adipose tissue: a three-way comparison of a research-specific, a freeware and a coronary application software platform

**DOI:** 10.1007/s00330-023-09878-5

**Published:** 2023-06-27

**Authors:** Jasmine Chan, Udit Thakur, Sean Tan, Rahul G. Muthalaly, Harsh Thakkar, Vinay Goel, Yeong-Chee Cheen, Damini Dey, Adam J. Brown, Dennis T. L. Wong, Nitesh Nerlekar

**Affiliations:** 1 Monash Cardiovascular Research Centre, Monash University and MonashHeart, Monash Health, Clayton, VIC Australia; 2 Cedars Sinai Medical Center, Biomedical Imaging Research Institute, Los Angeles, CA USA; 3 Baker Heart and Diabetes Institute, Melbourne, VIC Australia

**Keywords:** Adipose tissue, Atherosclerosis, Computed tomography angiography, Coronary artery disease

## Abstract

**Objectives:**

Epicardial adipose tissue (EAT) is a proposed marker of cardiovascular risk; however, clinical application may be limited by variability in post-processing software platforms. We assessed inter-vendor agreement of EAT volume (EATv) and attenuation on both contrast-enhanced (CE) and non-contrast CT (NCT) using a standard coronary CT reporting software (Vitrea), an EAT research-specific software (QFAT) and a freeware imaging software (OsiriX).

**Methods:**

Seventy-six consecutive patients undergoing simultaneous CE and NCT had complete volumetric EAT measurement. Between-software, within-software NCT vs. CE, and inter- and intra-observer agreement were evaluated with analysis by ANOVA (with post hoc adjustment), Bland-Altman with 95% levels of agreement (LoA) and intraclass correlation coefficient (ICC).

**Results:**

Mean EATv (freeware 53 ± 31 mL vs. research 93 ± 43 mL vs. coronary 157 ± 64 mL) and attenuation (freeware  − 72 ± 25 HU vs. research  − 75 ± 3 HU vs. coronary  − 61 ± 10 HU) were significantly different between all vendors (ANOVA *p* < 0.001). EATv was consistently higher in NCT vs. CE for all software packages, with most reproducibility found in research software (bias 26 mL, 95% LoA: 2 to 56 mL), compared to freeware (bias 11 mL 95% LoA: − 46 mL to 69 mL) and coronary software (bias 10 mL 95% LoA: − 127 to 147 mL). Research software had more comparable NCT vs. CE attenuation (− 75 vs. − 72 HU) compared to freeware (− 72 vs. − 57 HU) and coronary (− 61 vs.  − 39 HU). Excellent inter-observer agreement was seen with research (ICC 0.98) compared to freeware (ICC 0.73) and coronary software (ICC 0.75) with narrow LoA on Bland-Altman analysis.

**Conclusion:**

There are significant inter-vendor differences in EAT assessment. Our study suggests that research-specific software has better agreement and reproducibility compared to freeware or coronary software platforms.

**Key Points:**

*• There are significant differences between EAT volume and attenuation values between software platforms, regardless of scan type.*

*• Non-contrast scans routinely have higher mean EAT volume and attenuation; however, this finding is only consistently seen with research-specific software.*

*• Of the three analyzed packages, research-specific software demonstrates the highest reproducibility, agreement, and reliability for both inter-scan and inter-observer agreement.*

## Introduction

Epicardial adipose tissue (EAT) has been widely studied as a marker of cardiovascular risk across multiple domains including coronary artery disease [1], myocardial function [2] and cardiac dysrhythmia [3]. It has also been investigated in systemic disorders, particularly those pertaining to inflammation including chronic rheumatic, pulmonary and renal diseases [4, 5]. Whilst several studies have utilized echocardiographic linear thickness measures for EAT assessment, this has demonstrated suboptimal reproducibility and agreement with full volumetric assessment as assessed by computed tomography (CT) [6].

Volumetric EAT has been derived from both non-contrast (NCT) and contrast-enhanced (CE) studies that most often employ a lower threshold of  − 190 Hounsfield units (HU) and upper threshold of  − 30 HU to define adipose tissue within a contoured region. However, discrepancies are seen in absolute volumes with smaller values in CE, possibly due to the effects of contrast blooming and adjacent calcification that can cause partial volume artefact in contrast-enhanced scans [7]. An additional cause for discrepancy between scan modalities that has not been investigated is the influence of post-processing software packages.

As EAT is not routinely reported in clinical studies, different software have been employed from bespoke research programs, to extending the use of coronary artery reporting platforms, or manipulating plugins on Digital Imaging and Communications in Medicine (DICOM) image viewing software. This lack of standardisation may significantly influence interpretation and robustness of EAT as a cardiovascular risk marker. There is no gold standard for EAT measurement, likely due to difficulty in obtaining exact autopsy volumes of EAT in ex vivo models due to its extreme adherence to the underlying myocardium [8]. As EAT volume as well as attenuation can be measured which in effect represents the quantity and quality of adipose tissue, it is important to identify a high-fidelity post-processing software that appropriately encompasses both these parameters.

We therefore sought to assess inter-vendor differences and agreement in EAT measurement volumes and attenuation in patients who had simultaneous CE and NCT using an EAT research-specific software, a traditional coronary CT reporting software and a free DICOM imaging software.

## Materials and methods

We retrospectively studied 76 consecutive patients with moderate cardiovascular risk profiles as assessed on CAD Consortium Clinical Score who had simultaneous CE and NCT indicated for suspected coronary artery disease (July 2018). Patients with previous known cardiac intervention or cardiac prostheses were excluded to allow for a primary preventative screening population.

All CTs were performed using a Siemens Definition AS + (128 slice, Siemens Medical Solutions). NCT scans were performed prior to CE studies for each patient. Each CT was obtained in a single breath-hold and extended from the aortic arch to the diaphragm. Beta-blockers were administered prior to scanning to achieve the target heart rate of 60 beats per minute. The entire heart was scanned using prospective electrocardiograph-gating with tube modulation technique (120 kV; 280–350 mAs; pitch of 0.18 and 300 ms gantry rotation time). Both NCT and CE images were acquired at 0.6-mm slice thickness at 0.3-mm increments and reconstructed using a medium smooth kernel (B26, Siemens Medical Solutions).

CE studies were performed with injection of non-ionic iodinated contrast agent, iopromide (Ultravist 370 mg/mL, Bayer Healthcare) in an antecubital vein by a dual injector (Medrad Stellant). Individualized weight-based contrast volumes were injected at 6 mL/s in a triple phase pattern of pure contrast/50:50 saline mix/saline. Nitroglycerin was administered sublingually 1 min before contrast injection. A contrast bolus monitoring technique evaluating time to peak enhancement in the descending aorta was used to determine the scan delay (tube voltage 100 kVp and 20 mAs).

**Fig. 1:**
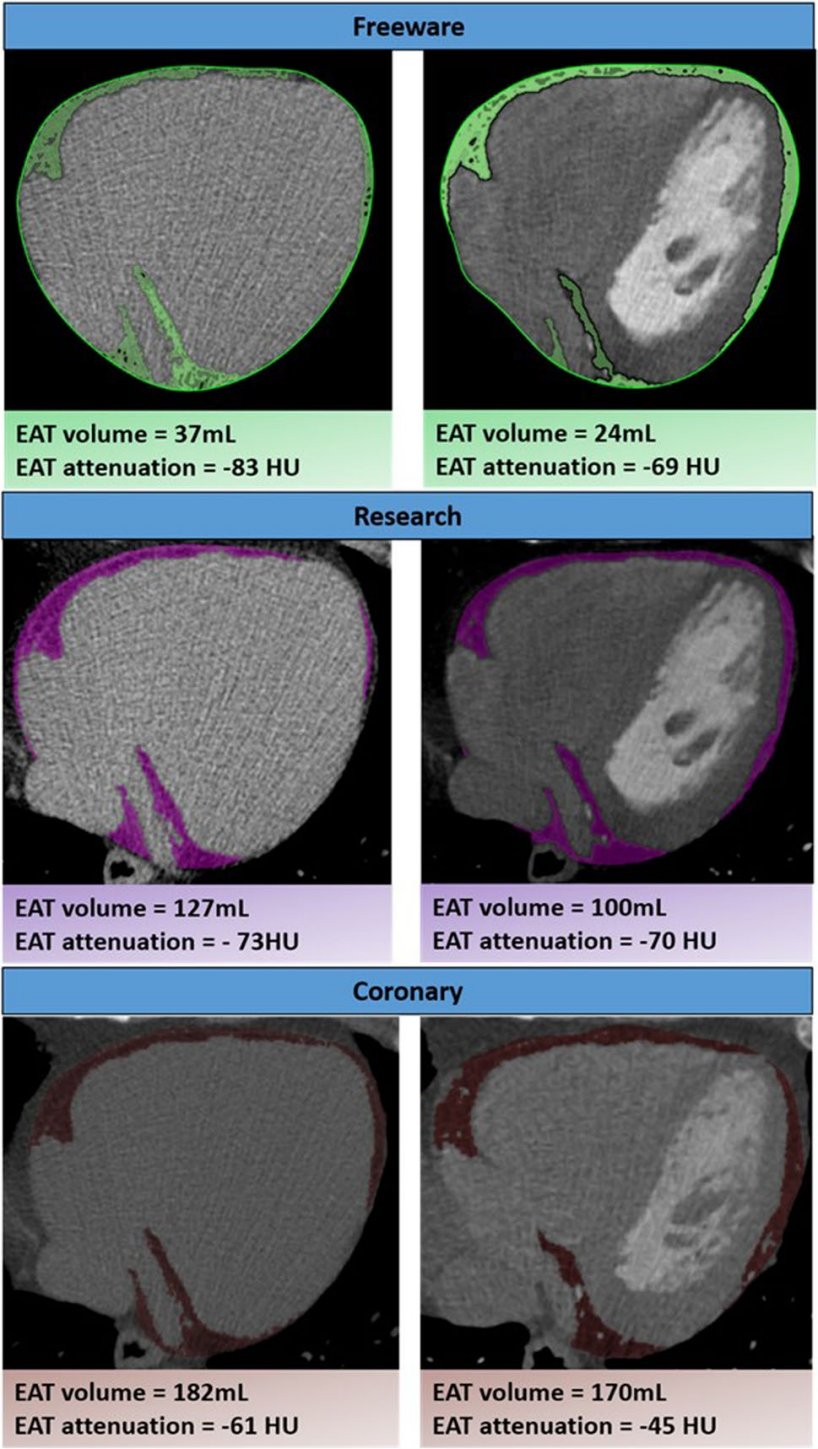
Inter-vendor comparison of EAT volume and attenuation in non-contrast (left) and contrast-enhanced (right) images by freeware, research and coronary software platforms. This demonstrates marked differences in calculated volumes despite similar visual appearance. EAT, epicardial adipose tissue; mL, millilitres; HU, Hounsfield units

We used three previously published software packages for EAT assessment: an EAT-specific software designed for research use, QFAT 2.0; a high-quality software specifically designed for coronary CT assessment, Vitrea 6.7 (Vital Images); and a free widely used DICOM software, OsiriX MD 9.0.2 (Pixmeo SARL). NCT and CE scans were anonymized and assessed by two independent, experienced imaging cardiologists in a blinded, randomized order of each different software package at different time points weeks apart to assess for inter-observer agreement. EAT areas were manually contoured for each scan across software platforms by each blinded assessor. For all EAT assessments, the upper and lower boundaries were considered to be the bifurcation of the pulmonary trunk and the lower most portion of the apex of the heart where the posterior descending artery could be seen. Individual software examples on the same patient are demonstrated in Fig. 1.

EAT volumes are presented as absolute volume measurements. EAT attenuation is presented as the mean attenuation of the entire EAT volume with standard deviations (SD). Data distribution was assessed for normality with the Shapiro-Wilk test. Statistical tests of inference were performed with *t*-tests or ANOVA as appropriate and Scheffe’s method for post hoc analysis. Bland-Altman graphs of the difference in the average of measurements with 95% limits of agreement were plotted to assess agreement between methods. Additional statistical assessment of inter-observer agreement was performed with the intraclass correlation coefficient. Analysis was performed with Stata MP14 (StataCorp) and GraphPad Prism (GraphPad Software). Ethics approval was obtained from the local human research ethics committee.

## Results

All patients had satisfactory image quality for assessment of EAT volume. The demographics of included patients are summarized in Table 1.

**Table 1:** Patient demographics

Age (years)	58 ± 15
Male gender (%)	56%
Hypertension (%)	48%
Hyperlipidaemia (%)	45%
Diabetes (%)	24%
Smoking (%)	36%
BMI kg/m^2^	28 ± 4
No coronary atheroma (%)	15%
Non-obstructive < 50% stenosis (%)	59%
Obstructive ≥ 50% stenosis (%)	26%

Data presented as percentage of the whole population or mean ± standard deviation

Stenosis is at a patient level

*BMI*, body mass index

### EAT volume by software type

**Fig. 2:**
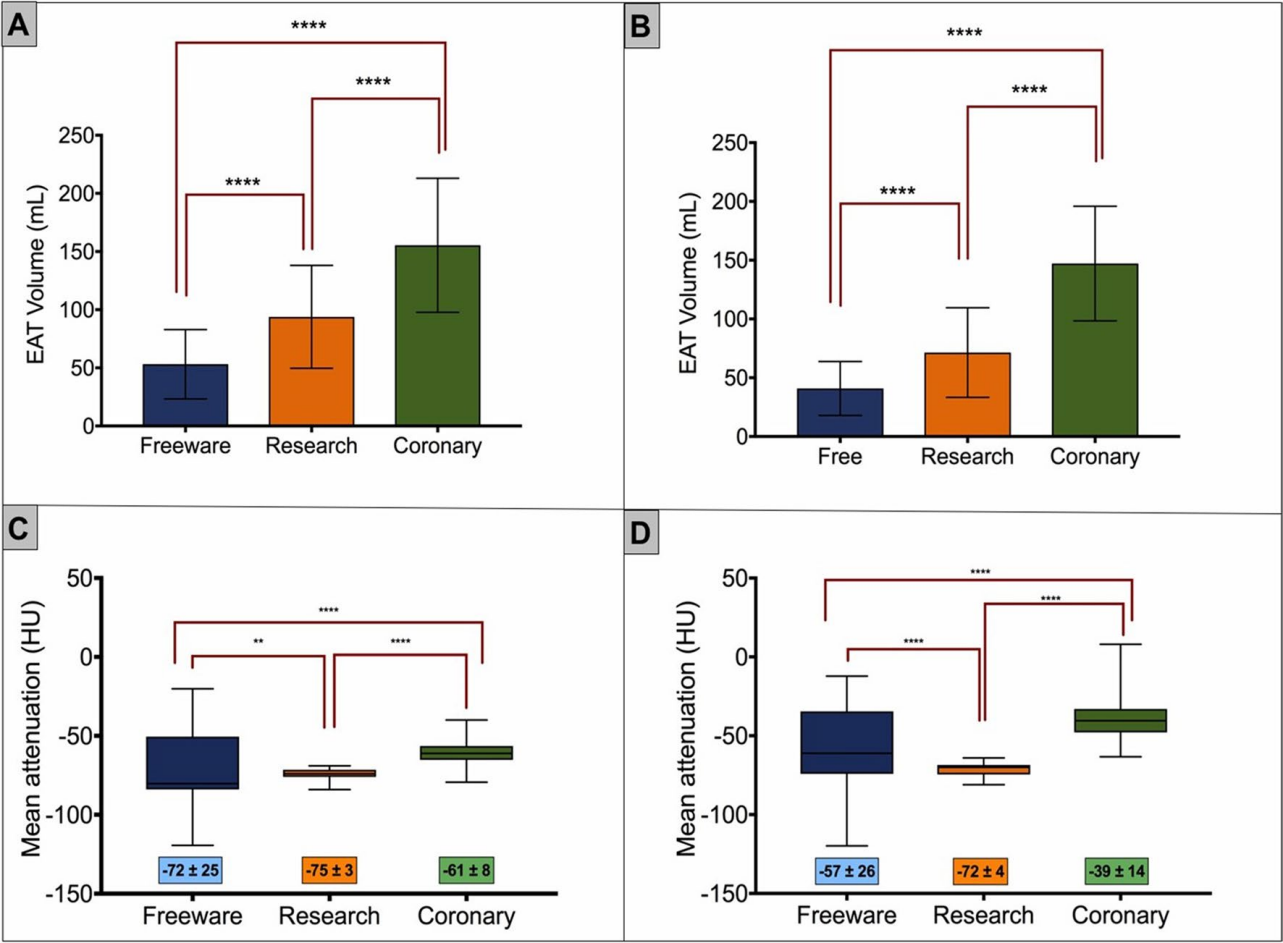
Differences in EAT volumes and attenuation for non-contrast (**A**, **C**) and contrast enhanced (**B**, **D**). There are significant volumetric and attenuation differences seen between each software platform overall and with pairwise comparison. Notably, the standard deviation for EAT attenuation with research software is narrower compared to other software. EAT, epicardial adipose tissue; HU, Hounsfield units; mL, millilitres

There were significantly different measures of mean EAT volume between each software platform for both NCT and CE scans. Mean NCT EAT volume by freeware software was 53 ± 31 mL, 93 ± 43 mL for research software and 157 ± 64 mL for coronary software (ANOVA *p* < 0.001). Similarly, significant differences were noted for CE datasets: freeware 41 ± 23 mL vs. research 71 ± 38 mL vs. coronary 147 ± 50 mL (*p* < 0.001) (Fig. 2, Table 2). Significant differences were seen between individual comparisons of volumes between each software type on post hoc testing.

**Table 2:** Summary of CT EAT volume and attenuation

	Freeware	Research	Coronary	*p*-value
NCT				
Volume (mean ± SD) (mL)	53 ± 31	93 ± 43	157 ± 63	< 0.001
Attenuation (mean ± SD) (HU)	− 72 ± 25	− 75 ± 3	− 61 ± 8	0.001
CE				
Volume (mean ± SD) (mL)	41 ± 23	71 ± 38	147 ± 50	< 0.001
Attenuation (mean ± SD) (HU)	− 57 ± 26	− 72 ± 4	− 39 ± 14	< 0.001

*p*-value for trend by ANOVA

### EAT attenuation by software type

There were significant differences noted with EAT attenuation values between platforms for NCT. Research software demonstrated the narrowest range and SD with mean − 75 ± 3 HU compared to coronary software (mean − 61 ± 10 HU) and freeware software (mean − 72 ± 25 HU) (ANOVA *p* < 0.001). Similar differences were also noted between software platforms for CE scans, with research software demonstrating the narrowest range and SD (mean − 72 ± 4 HU) when compared to coronary software (mean − 39 ± 14 HU) and freeware software (mean − 57 ± 26 HU) (ANOVA *p* < 0.001) (Fig. 2, Table 2). There were significant differences between groups for both NCT and CE scans on post hoc testing.

### Inter-scan and within software variability

**Fig. 3:**
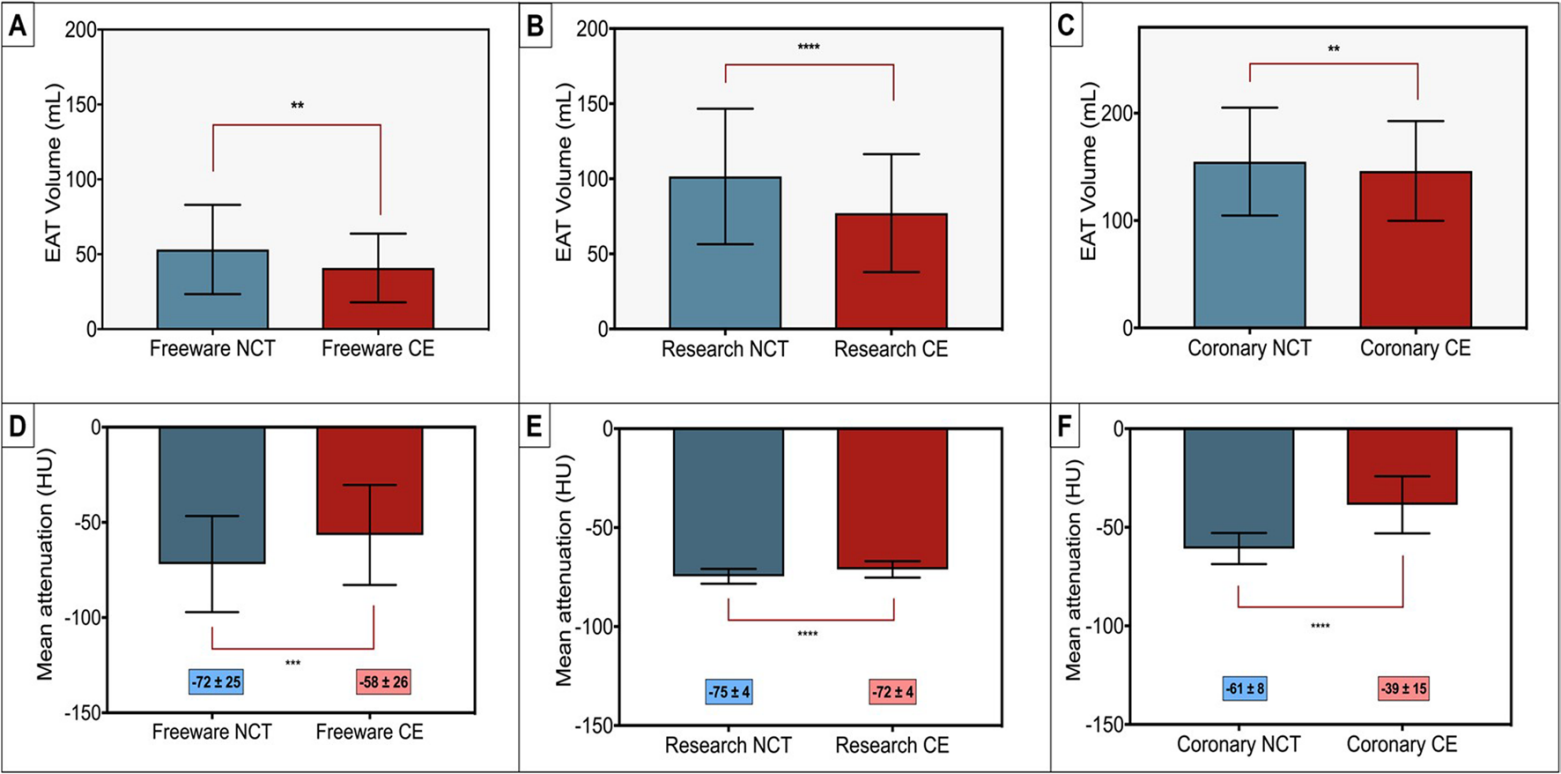
Inter-scan differences by software type. EAT volume by non-contrast vs. contrast (**A**–**C**) and attenuation (**D**–**F**) are depicted. This demonstrates that there are significant differences between NCT and CE regardless of software with a smaller volume and higher attenuation on contrast scans. However, absolute differences with research software attenuation is low (− 3 HU) compared to  − 14 HU for freeware and  − 21 HU for coronary software. CE, contrast enhanced; EAT, epicardial adipose tissue; HU, Hounsfield units; mL, millilitres; NCT, non-contrast CT

**Fig. 4:**
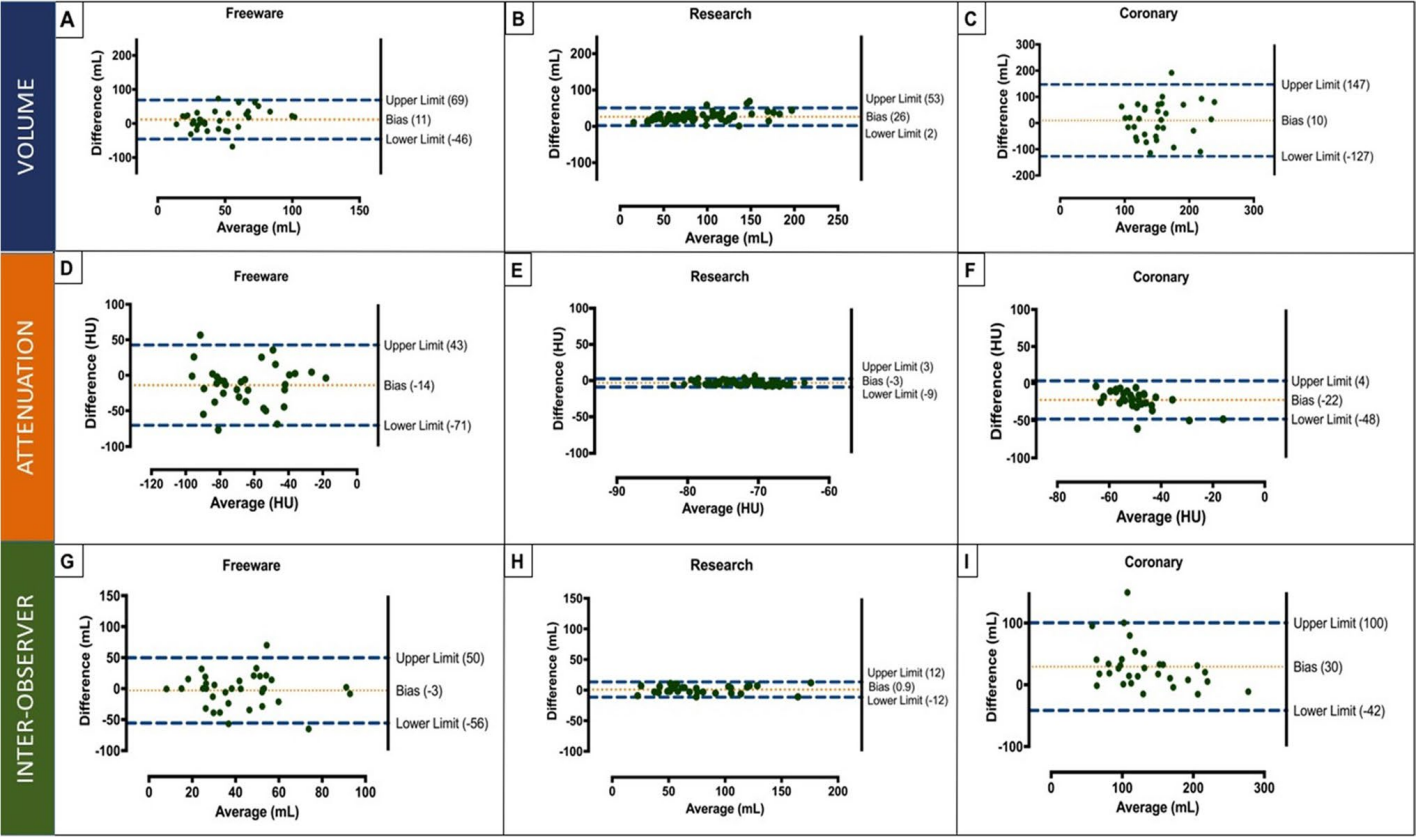
Bland-Altman plots for inter-scan differences by volume and attenuation and inter-observer variability by software type (freeware—left panel; research—middle panel; coronary—right panel). This visually demonstrates high levels of agreement for research software (all *y*-axis scales are consistent). Importantly, NCT consistently had a higher volume than contrast only with research software (mean difference 26 mL) with over- and underestimation on freeware and research software. HU, Hounsfield units; mL, millilitres; NCT, non-contrast CT

EAT volume and attenuation were noted to be higher in NCT compared to CE scans across all software platforms (Fig. 3). Research software demonstrated a higher volume on NCT scans (bias 26 mL) with 95% lower bound limit of agreement 2 mL, with similar higher EAT volumes on freeware and coronary software but smaller bias values and markedly variable lower bound limits of agreement (freeware bias 11 mL, 95% lower LoA − 46 mL; coronary bias 10 mL, 95% lower LoA − 127 mL). Attenuation was also higher in NCT compared to CE scans across all software packages, but the absolute differences in attenuation between both scans were significantly lower with research-specific software (mean difference 3 ± 3 HU) compared to freeware (14 ± 29 HU) and coronary software (22 ± 13 HU). Bland-Altman plot analysis demonstrated a very high level of agreement with research software for both EAT volume and attenuation (Fig. 4) with no specific visual systematic differences.

### Inter-observer agreement

There was an excellent level of agreement of EAT volume with research software with an intraclass correlation coefficient of 0.98 (95% CI 0.96–0.99, *p* < 0.001). Moderate agreement was noted with coronary and freeware software, although not as high as research-specific software: coronary ICC 0.75 (95% CI 0.55–0.87, *p* < 0.001); freeware ICC 0.73 (95% CI 0.38–0.88, *p* < 0.001). Bland-Altman plots similarly demonstrated a high level of agreement for research software with narrow limits of agreement (bias 0.9 mL, 95% LoA − 12 to 12 mL) compared to freeware (bias − 3 mL, 95% LoA − 56 to 50 mL) and coronary software (bias 30 mL, 95% LoA − 42 to 100 mL). Similarly, high levels of agreement were seen for attenuation with research software with excellent agreement (ICC 0.95 (95% CI 0.92–0.99)) and poor agreement for freeware (ICC 0.52 (95% CI 0.03–0.76)) and coronary software (ICC 0.54 (0.06–0.78)) (Table 3, Fig. 4).

**Table 3:** Observer variability for inter- and intra-observer differences

	Freeware			Research			Coronary		
	Bias	95% LoA	ICC (95% CI)	Bias	95% LoA	ICC (95% CI)	Bias	95% LoA	ICC (95% CI)
Volume	11	− 46 to 69	0.73 (0.38–0.88)	26	2 to 53	0.98 (0.96–0.99)	10	− 127 to 147	0.75 (0.55–0.87)
Attenuation	− 14	− 71 to 43	0.52 (0.03–0.76)	− 3	− 9 to 3	0.95 (0.92–0.99)	− 22	− 48 to 4	0.54 (0.06–0.78)

Data from non-contrast CT

## Discussion

This is the first study of an inter-software and inter-scan comparison of EAT volume and attenuation measurements utilising a research-specific, coronary-specific and freeware DICOM processing software. Our main findings can be summarized as follows: (1) Calculated EAT volumes differ significantly between software programs regardless of scan type; (2) NCT scans have higher mean EAT volume and attenuation compared to CE scans; (3) Research-specific software has the highest reproducibility and inter-observer agreement compared to a freeware and a coronary-specific software program. In the absence of a standardized method for assessing EAT volume and attenuation, this suggests that a robust research-specific software is the most reliable platform for analysis of EAT.

### Epicardial fat measurement

EAT is the natural visceral adipose tissue of the heart beneath the visceral pericardial layer and the myocardium. Paracardiac fat is situated above the fibrous pericardial layer. Together, paracardiac and epicardial fat are often regarded as pericardial adipose tissue [9]. Although pericardial adipose tissue as a whole has previously been shown to correlate with other cardiovascular markers such as coronary artery calcium score [10], each of its components, EAT and paracardiac fat, is highly unique with different embryologic origins and blood supply. Individually, EAT has been more linked with the development of cardiac disease due to its idiosyncratic location. EAT functions as an endocrine organ with pro-inflammatory mediators that may infiltrate into the neighbouring coronary arteries and myocardium resulting in pathologic dysfunction [11]. EAT is also proposed to have protective benefits, by providing a cushioning support for the coronary arteries and as an energy store in times of cardiac stress [12]. As all patients have some degree of EAT, it may in fact not be the quantity but rather the quality of EAT that leads to disease. Recent evidence suggests that EAT attenuation may be a marker of adipose dysfunction and has shown promise in refining the role of EAT in cardiovascular disease [13–17]. Higher, or more ‘negative’ HU, values suggest greater lipid content and more ‘positive’ values suggest higher water content, or alternatively, smaller and less mature lipids [18]. It has been suggested that inflammation is a driving force for arrest of lipid maturation [19], and therefore, EAT may serve as a marker of adjacent vascular inflammation [20]. Conversely, larger lipid content may result in a greater proportion of dysfunctional adipokines that can cause vascular and metabolic damage [21]. This bidirectional communication of cytokines is a principal hypothesis for the pathologic relationship between EAT and cardiac disease [22]. This may suggest a dynamic relationship between inflammation and EAT, leading to atherogenesis and alteration in plaque morphology, a marker of poor cardiovascular outcomes [23].

Although its clinical significance, there has been difficulty in determining a gold standard method to evaluate EAT. EAT is immediately apposed and extends into the underlying myocardium without any fascial separation [24]. EAT is also differentially distributed around the heart with the greatest volumes seen around the right ventricle [25]. Therefore, the ability to adequately excise and measure EAT at autopsy is technically challenging, with potential contamination from adjacent paracardiac fat as an additional confounder. As such, no large systematic studies have been performed to compare post-mortem and radiographic EAT volume. It is for this reason that there remains no consensus on population thresholds for EAT measurements and reliance is placed on radiographic imaging techniques to evaluate EAT. This lack of a gold standard for EAT assessment has resulted in variable reported volumes from imaging studies which has decreased generalisability and clinical utility of this parameter.

### Inter-software volume and attenuation

In our study, we demonstrated marked differences in EAT volume assessed between software programs. This important finding reflects the uncertainty of a generalizable threshold for EAT volume that associates with disease with several suggested cutoff values within the literature [1] given the lack of an anatomical gold standard. Similarly, significant differences were seen with mean HU attenuation. Our findings have mirrored previous studies by demonstrating lower SD of attenuation with research software (± 3 HU) compared to other software (coronary ± 10 HU and freeware ± 25 HU) in both NCT and CE scans, suggesting better reproducibility (standard error 0.6HU) [14, 26].

The differing results between software packages are unclear; however, we speculate that this could be due to the inherent differences in coding and programming in each software package. The research software utilized, QFAT 2.0, was specifically designed with in-built filters, reconstruction algorithms and enhanced automation for the purpose of EAT assessment. The program has been repeatedly refined with automated deep learning techniques to reduce variability in measurement [27], whilst the other software platforms were not. Coronary software was modified to assess EAT rather than its original purpose of assessing coronary plaque, whilst the freeware software used was a generic image viewing platform that was manipulated to evaluate EAT. Hence, the different coding for each software could explain the differing EAT volume and attenuation results, low SD for attenuation with research software, and reproducibility found in this study. Nevertheless, it is important to note that these results only reflect better replicability, rather than true accuracy, of EAT volume and attenuation with research software, given the lack of an anatomical gold standard for comparison.

### Inter-scan volume and attenuation

We noted significant differences in EAT volume between scan types, with higher volumes measured on NCT scans. Research software consistently demonstrated higher NCT volumes (mean difference 26 mL) with a lower 95% limit of agreement of 2 mL with no cases of discordance, whilst freeware and coronary software also had positive mean differences, indicating a general disposition of higher NCT EAT volumes but comparative lower 95% limits of agreement of  − 46 mL and − 127 mL respectively. This finding has been previously reported in similar small studies [7, 28]. This is not a surprising finding and can be explained by partial volume artefact from luminal contrast enhancement, image interpolation and differing spatial resolution, as well as potential vascularity of adipose tissue [29]. Given the lack of a gold standard measure and anatomical correlation, it is unclear which software measurements most reflect true EAT volume on NCT scans, but this finding is suggestive that research software volume measurements are more reproducible.

Marked differences were noted between NCT and CE mean attenuation values for freeware and coronary software, with nominally small absolute difference for research software, despite a significant difference between scan type reflective of the narrow data spread. CE significantly lowered the mean attenuation in all software programs, although the absolute difference with research software was low at 3 HU. The marked differences in non-research software accompanied by their wide dispersion of data reduce reliability of these post-processing methods.

### Inter-observer agreement

Whilst most EAT studies do report inter- and intra-observer agreement for volume measures, no study has compared differences in post-processing software. We show that inter-observer agreement was near perfect with research software with very narrow limits of agreement on the Bland-Altman plot. Whilst good agreement was still demonstrated with coronary and freeware software based on the intraclass correlation coefficient, a wider dispersion of data points was visually noted on Bland-Altman plot with broad limits of agreement. There is inherent appeal to extend the use of a standard coronary reporting platform or a freely available image processing software rather than a dedicated research software which requires additional time for measurement. However, similar to any diagnostic tool, rigorous assessment is required before any software can be considered satisfactory for clinical application. Our findings suggest that software variability may explain some of the contrasting results seen in epicardial fat research. The research software employed has been rigorously tested for its application of adipose tissue measurement. In its inception, individual voxel limits pertaining to adipose tissue were determined from training datasets and then applied to epicardial adipose tissue boundaries by segmentation of connected voxels within a defined CT attenuation range (− 190 to  − 30 HU) [30]. This technique has been further refined with serial improvements and image filtering to a point of complete automation with deep learning techniques [27]. The technique and calculation algorithms of other software are not readily available and do not have as substantial a publication background.

### Limitations

We acknowledge several limitations with our study. Firstly, we have tested only three of the multiple software packages available for EAT measurement and our results may not be germane to other platforms. Additional analysis with other software is required to assess if our results hold true. Secondly, as mentioned, given the lack of a reference standard for EAT volume, we instead focused on agreement and reproducibility of sample estimates to guide a more robust radiographic tool which arguably may be superior to autopsy sampling given the challenge of EAT dissection. We did not assess the accuracy of the different software platforms against other markers for cardiovascular disease such as traditional cardiovascular risk factors and coronary artery calcium score. Thirdly, we were unable to confirm the differences in volumetric measurements between software packages by measuring volumes of alternative cardiac structures, as the research software could not be modified to assess non-EAT volumes. Additionally, our sample size is small and heterogeneous. Finally, we did not have access to underlying algorithms for fat segmentation in each software package and therefore cannot mathematically explain our results or provide a correction factor.

## Conclusion

We found significant differences in inter-platform EAT assessment for both volume and attenuation measures. Research-specific software appears to have higher level of agreement and reproducibility compared to the tested freeware and coronary software platform and may be the preferable tool for EAT assessment in future studies.

## Abbreviations

CE: Contrast enhanced
CT: Computed tomography
EAT: Epicardial adipose tissue
HU: Hounsfield Units
LoA: Limits of agreement
NCT: Non-contrast CT

## Declarations

### Guarantor

The scientific guarantor of this publication is Dr Nitesh Nerlekar.

### Statistics and biometry

One of the authors has significant statistical expertise.

### Study subjects or cohorts overlap

No study subjects or cohorts have been previously reported.

### Methodology

• retrospective

• cross-sectional study

• performed at one institution
